# Ascertaining Consciousness in Identity Formation: Best Medical Practices in Dementia Care

**DOI:** 10.1093/geroni/igaa057.1876

**Published:** 2020-12-16

**Authors:** Angel Duncan

**Affiliations:** Albertus Magnus College, New Haven, Connecticut, United States

## Abstract

This session identifies common misconceptions about identity for persons living with Alzheimer’s disease and related dementias (ADRD). Going beyond diagnostic brain imaging and neurocognitive testing, case studies and research in creativity from around the United States highlights consciousness of persons living with ADRD. Reviewing and discussing artworks is aimed to set dialogue in the question of where memory deposits emerge when engaged in creativity. Through art therapy techniques, this type of self-expression may provide new avenues in treatment for dementia care. Exploring the arts from those with Mild Cognitive Impairment to late stage Alzheimer’s and other forms of dementia, such as frontotemporal dementia, consciousness seems to remain intact despite neural death. This session aims to discourage poor spending allocations and establishing meaningful care. From clinical research trials to creativity of self-expression, the importance of why the arts and sciences matter are demonstrated as effective modalities that enhance quality of life.

